# Biostimulators with marine algae extracts and their role in increasing tolerance to drought stress in highbush blueberry cultivation

**DOI:** 10.1371/journal.pone.0306831

**Published:** 2024-09-19

**Authors:** Agnieszka Lenart, Dariusz Wrona, Tomasz Krupa

**Affiliations:** Department of Pomology and Horticultural Economics, Warsaw University of Life Sciences - SGGW, Warszawa, Poland; Bahauddin Zakariya University, PAKISTAN

## Abstract

Drought is one of the most serious challenges facing agriculture and ecosystems around the world. With more frequent and more extreme weather events, the effects of drought are becoming more severe, leading to yield losses, soil depletion and environmental degradation. In this work, we present an analysis of the impact of a marine algae biostimulanat andits ability to offset the effects of drought stress in blueberry cultivation. The aim of the research was to evaluate various fertilisation programs in increasing plant resistance to abiotic stress such as drought. It was tested whether the algal biostimulator provides the same tolerance to drought stress in highbush blueberry plants as regular fertilisers without biostimulation. The research was conducted in 2022 in a greenhouse in controlled drought conditions. Three-year-old highbush blueberry bushes (12 pieces) were used in the experiment. Highbush blueberry bushes (*Vaccinium corymbosum*) ’Brigitta Blue’ varieties were planted in plastic pots with a capacity of 10 dm^3^ containing an acidic substrate and placed in a greenhouse. Controlled lighting conditions were maintained using sodium lamps and a temperature of 25°C/20°C day/night. The substrate in pots was maintained at 80% of field water capacity by manual watering and weekly supply of nutrient solution for 5 weeks until water deficit occurred. Half of the plants were sprayed weekly with biostimulant at a concentration of 1%, three times 1 week apart (1 application per week). The biostimulant was evenly applied to the entire plant. Seven days after the third application of the product, half of the unsprayed and sprayed plants were subjected to water deficit stress by holding thewatering until 40% of the field water capacity (FC) was reached. The experimental layout included four combinations: C—Control—no biostimulation, no water deficit; CS—Stress control—water deficit up to 40% FC, no biostimulation; B—Biostimulator—no water deficit, biostimulation; BS—Stress plus biostimulator—water deficit up to 40% FC, biostimulation. Fertilisers with seaweed extracts show the ability to reduce the adverse effects of stress, promoting plant resilience, including tolerance to drought stress. The following were evaluated in the experiment: catalase activity, peroxidase activity, free malondialdehyde content, photosynthetic activity and leaf mineral content. The biostimulant used in experiment increased the oxidative activity of the enzymes pe-roxidase and catalase under simulated drought stress conditions. The algal biostimulant increased the average value of catalase activity by 20% in comparison to the control plants, in both combinatinations. The tested biostimulator had no effect on the chlorophyll content in the leaves or the concentration of nutrients in the leaves. The effect of marine algae products on the yield quantity and high quality is related among other to bioactive substances which helps to prevent drought stress.

## Introduction

Crops are often exposed to various negative environmental influences [1]. Abiotic stress is the cause of huge losses in agriculture [2, 3]. Rapidly progressing climate changes cause long periods of drought, which contribute to significant losses in plant production [4]. Lack of water in the soil negatively affects the uptake of minerals, limits plant growth and crop efficiency [5–7]. Compared to other abiotic stressors, drought causes significant losses in crop production and affects global food security [8–10]. The negative impact of abiotic stress can generate up to 70% yield loss [11, 12]. Rational water management in agriculture and the search for drought-resistant varieties are an important factor in preventing crop losses [13]. Breeding varieties genetically resistant to drought stress is difficult and time-consuming, therefore alternative solutions to increase plant tolerance to unfavourable environmental conditions are still being sought [14, 15]. The highbush blueberry is a species very sensitive to water shortages due to its shallow root system, penetrating the soil to a depth of 40 cm [16]. Even a short period of water shortage causes a stress reaction in the plant, limiting photosynthesis and inhibiting plant growth [17, 18]. The demand for highbush blueberry fruit is still growing and the profits from cultivation encourage growers to increase the cultivated areas of this species [19, 20]. Blueberries are considered naturally healthy and valuable fruits, containing a number of various bioactive compounds that have a positive effect on human health [21]. These fruits are a valuable source of compounds such as fibre, minerals (potassium, manganese, iron, zinc, calcium, magnesium, and phosphorus), vitamins (C, K, A, E, and B vitamins), and phenolic compounds with antioxidative properties (anthocyanins, flavanols, ellagic acid) [22].

In the modern cultivation of highbush blueberries, it is important to use innovative technologies that allow the production of more good quality fruit [16]. One of the ways to increase the resistance of plants to biotic and abiotic stresses may be the use of biostimulants [23]. Plant biostimulants are defined as products containing various bioactive organic or inorganic substances that are able to stimulate plant growth, yield and eliminate the adverse effects of abiotic stresses [24, 25]. Biostimulating compounds improve soil conditions and, indirectly, also affect the physiology and metabolism of plants [26]. Plants treated with seaweed extracts take up, assimilate and feed nutrients faster compared to untreated plants, have stronger growth and better developed root system capable of more extensive nutrient uptake [26]. The synergistic effect of technology with biostimulation, based on seaweed extracts, on the percentage of large fruit and on qualitative yield was shown by Kaplan et al [27]. An experiment conducted by Lenart et al [21] showed the algal fertilizers increase the antioxidant activity of highbush blueberry fruit, the content of anthocyanins and total polyphenols, which is related not only into better fruit health promoting properties but also into better storage. Algae biostimulants also influence processes at the cellular level, such as: the synthesis of osmoregulatory compounds, the activation of antioxidant enzymes, the ability to accumulate water in plant tissues and the stimulation of cell division [28, 29]. According to Van Oosten et al. [30] biostimulants influence the better use of water and minerals by plants, support their development and counteract abiotic stresses. The key response of plants to drought stress is the production of substances such as osmolytes, osmoprotectants and antioxidants [31]. It was found that when plants are exposed to abiotic stress, in plant cells occurs an increase in the production of H_2_0_2_ [32, 33]. Reactive oxygen species (ROS) are a key element of the network of signalling pathways and play a significant role in the metabolism and aging of plant cells [32, 34]. In low concentrations H_2_0_2_ acts as a signalling molecule involved in the regulation of specific physiological processes such as photosynthesis, plant growth and development, cell cycle and plant responses to stress [35]. An important element of the plant’s response to the accumulation of ROS caused by a stressful situation is the activity of oxidizing enzymes such as catalases or peroxidases [36, 37].

The present work was carried out within the framework of the project "Implementation PhD" (contract no. 0060/DW/2018/02) organised by the Ministry of Science and Higher Education.

The aim of the project is to implement the developed solutions in real conditions or industrial environments. The study analysed whether a biostimulant preparation containing marine algae extracts reduces abiotic stress on plants caused by water shortage. A parallel aim of the research was to prepare an argumentation supported by experimental results with a view to marketing the algae preparation. The research hypothesis is that the tolerance of highbush blueberry plants to drought stress increases following the application of marine algae sourced biostimulant compounds.

The purpose of the research was to evaluate various fertilisation programs and their role in increasing plant resistance to abiotic stress such as drought. The aim of the experiment was to examine the differences between fertilisation with and without biostimulation.

## Materials and methods

### Location and layout of the study

The work was carried out as part of the program of the Ministry of Science and Higher Education "Implementation Doctorate" no. 0060 / DW / 2018/02. The experiment as a part of the research cycle was conducted in 2022 in the controlled greenhouse conditions of the CMI Global Innovation Center in Saint Malo, France.

Three-year-old highbush blueberries were used in the experiment (12 plants). Blueberry plants (*V*. *corymbosum* ’Brigitta Blue’) were transplanted in 10 dm^3^plastic pots containing an acidic substrate (sphagnum peat moss without fertilisers, pH (H2O) 3.5–4.5, Klasmann-Deilmann GmbH, Geeste, Germany), placed in the greenhouse with a 16h/8h (day/night) photoperiod, controlled by supplementary lighting from high-pressure sodium lamps, and a temperature of 25°C/20°C day/night. Pots was maintained at 80% of field capacity by manual watering and weekly supplied with the following nutrient solution (Table 1) for 5 weeks until the triggering the water deficit.

**Table 1:** Composition of the nutrient solution [38].

Nutrient solution used for watering		
Element	Product	element per pot and per supply (g)
N	Ammonium sulfate	0.307
P_2_O_5_	Potassium phosphate	0.231
K_2_O	Potassium sulfate	0.538
Mg	MgSO_4_, 7H_2_O	0.153
Mn	MnSO_4_, H_2_O	0.006
Zn	ZnSO_4_, 5H_2_O	0.009
B	H_3_BO_3_	0.001
Cu	CuSO_4_, 5H_2_O	0.000
Fe	EDTA, 2NaFe, H_2_O	0.011

Half of the plants were weekly treated with a seaweed extract based biostimulant at 1% + 0.15% Heliosol three times with an interval of 1 week (1 application per week). The biostimulant was evenly applied on the whole plant until run out seven days after the 3rd product application, half of the untreated and treated plants were subjected to water deficit stress by stopping the watering for 5 days to reach 40% of field capacity (FC). We have four experimental groups::

1. C—Control—normal conditions (plant growing under normal conditions, without biostimulant and without water deficit)
2. CS—Control stress—water deficit conditions (40% FC; plant growing under water deficit condition, without biostimulant)
3. B—Biostimulant—normal condition with biostimulant (plant growing under normal conditions, with biostimulant application)
4. BS—Biostimulant stress—water deficit conditions (40% FC) with biostimulant (plant growing under water deficit condition, with biostimulant application).

According to the manufacturer’s description–company Timac Agro, the biostimulator used in the experiment (Kaoris) is an innovative biostimulating preparation based on plant extracts and brown algae extracts (Table 2).

**Table 2:** Composition of the biostimulating preparation.

Plant extracts, including marine algae	N	P	K	B	Cu	Mo
30%	3%	3%	9%	0,01%	0,002%	0,001%

Prior to triggering the water deficit (days 0), a random SPAD (MC-100 Chlorophyll Meter, Apogee Instruments, Inc.) measurements on three leaves per plant at mid-height have been performed. Then, 8 to 10 leaves per plant were sampled, frozen in liquid nitrogen then stored at -80°C until further analysis of the antioxidant activities in leaves. The same procedure (SPAD measurement and leaf sampling) has been followed at 2, 8, 12 and 15 days after triggering the water deficit (days 0). SPAD (Soil-Plant Analysis Development) is a measurement method used to assess the level of chlorophyll content in plant leaves. The measurement result is expressed in SPAD units.

### Catalase and peroxidase activity

#### Extraction

For enzymatic activity, 200 mg of powdered leaf tissue were extracted in 100 mM phosphate buffer-pH 7 supplemented with PVP. The extract was homogenised and followed by centrifugation at 12 000 g for 20 min at 4°C. The supernatant which constitutes the enzyme extract was collected in a new tube and used for colorimetric determination of catalase and peroxidase activity.

#### Catalase assay

50 μL of enzyme extract and 100 μL of 10mM hydrogen peroxide were added to a 96-well plate. The plate was vortexed and incubated at 37°C for 2 min, followed by the addition of a working solution consisting of cobalt (II), sodium hexametaphosphate and sodium carbonate. The plate was vortexed again and incubated at room temperature for 10min in the dark until the development of green colour. The changes in absorbance were recorded at 440 nm against the reagent blank. The dissociation of hydrogen peroxide is proportional to the activity of the catalase enzyme in the used sample [TK1] [AL2]. The results are given in μmol/min/g^-1^ FW.

#### Peroxidase assay

10 μL of enzyme extract and 100 μL of working solution (50 mM phosphate buffer, 20 mM hydrogen peroxide and 0.05% Guaiacol) were added to a 96-well plate. The changes in absorbance were recorded at 470 nm over a time interval of 2 mins (one reading every 15 sec). Peroxidase activity was measured as oxidation of guaiacol (ε = 26.6 mm–1 cm–1) in the presence of hydrogen peroxide within 2 mins [TK3] [AL4]. The results are given in μmol/min/g^-1^ FW.

### Malondialdehyde (MDA) measurement

100 mg of fresh ground tissue was extracted with 0.1% TCA extraction solvent and vortexed for 20 mins in cold (protected from light). The extract was centrifuged at 10000 g for 15 min at 4°C and the supernatant was set aside and filtered using a 0.2μm filter. The filtrate was transferred to a new tube and 1 mL of TBA (Thiobarbituric acid) reagent was added, mixed, and incubated for 15 min in a water bath at 95 °C, followed by 5 min incubation in ice. After centrifugation at 10,000 g for 10 min at 4°C, the supernatant was transferred to a 96-well plate and changes in absorbance were recorded at 440 nm, 532 nm, and 600 nm. The amount of the MDA–TBA complex was calculated using the extinction coefficient 155 mM–1cm–1. The results are given mg/g^-1^ FW.

### Elemental analysis

Analysis of total C, N and S was performed using an elemental FLASH 2000 CHNS analyser (Thermo Fisher Scientific, Waltham, MA, United States) according to the instructions of the manufacturer from 2.5 mg of homogenized and lyophilized plant material.

For other elements, the analysis was performed using inductively coupled plasma-optical emission spectrometry (ICP-OES, 5110 VDV, Agilent, CA, United States) with prior microwave acid sample digestion (Multiwave Pro, Anton Paar, Les Ulis, France) [8 ml of concentrated HNO3, 2 ml of H_2_O_2_, and 15 ml of Milli-Q water for 100 mg dry weight (DW)]. The quantification of each element was carried out with an external standard calibration curve.

The results are given mg/g^-1^ DW.

### Statistical analysis

Test results were analysed statistically using the one-way and two-way analysis of variance method. The inference was based on the significance level <0.05. All statistical analyses were performed in the SAS Enterprise Guide 5.1 program (Sas Institute Sp. z o.o., Warsaw, Poland).

## Results

The impact of the applied fertiliser combinations on the activity of catalase and peroxidase enzymes and the level of malondialdehyde (MDA) without the drought factor is presented in Table 3. The level of catalase activity in the study was significantly higher in combination B (Biostimulant) compared to combination C (Control) in all sampling days of the study, except for day 0. The activity of catalase in combination C increased significantly only on the 8th day of measurements. In combination B, catalase activity increased significantly on days 2, 8 and 12 of measurements. The fertiliser combinations used in the experiment did not affect the level of malondialdehyde (MDA) in the tested plant material (Table 3). In combination C, the MDA level was similar on individual test days, but on the 12th day of measurement, a significant decrease was noted. The MDA content in the leaves of plants from combination B differed significantly on different measurement days, the lowest value of the tested indicator was determined on the 12th day of the measurement, and the highest on day 0, measurements on days 2, 8 and 15 were characterized by intermediate values for the tested indicator. Peroxidase activity did not depend significantly on the fertiliser combinations used. No significant differences were noted between combinations C and B (Table 3). However, differences were found in both combinations C and B depending on the measurement day. In combination C, the lowest value of peroxidase activity was recorded on the 15th day of testing, and the highest after the 12th measurement day, while on the remaining days the peroxidase content was at a similar level. In combination B, lower peroxidase values were recorded on days 2 and 15, and higher values on other test days.

**Table 3:** Effect of fertilisation program on the activity of oxidizing enzymes and on the level of the oxidative stress marker malondialdehyde (MDA). Combinations without a drought factor.

	Combination	Sampling days					
		0	2	8	12	15	p-value
Catalase concentration (μmol/min/g^-1^ FW)	C	176,6 ^Aa^	201,8^Aa^	282,7^Ab^	163,6^Aa^	162.8^Aa^	0.0001
	B	182,4^Aa^	352,6^Bb^	347,4^Bb^	325,5^Bb^	219.5^Ba^	0.0001
	p-value	0,9733	0,0332	0,0155	0,01048	0.0461	
MDA concentration (mg/g^-1^ FW)	C	0,051^Ab^	0,057^Ab^	0,046^Ab^	0,033^Aa^	0.048^Ab^	0.0430
	B	0,048^Ab^	0,045^Aab^	0,042^Aab^	0,034^Aa^	0.037^Aab^	0.0767
	p-value	0,9581	0,2407	0,5517	0,5011	0.1344	
Peroxydase concentration (μmol/min/g^-1^ FW)	C	0,043^Ab^	0,047^Ab^	0,047^Ab^	0,067^Ac^	0.029^Aa^	0.0001
	B	0,036^Aa^	0,063^Ab^	0,032^Aa^	0,055^Ab^	0.021^Aa^	0.0013
	p-value	0,1641	0,8611	0,0671	0,0719	0.8391	

Capital letters in the column are for comparing the Modalities, small letters in the line are for comparing the impact of particular fertilisation programmes.

The impact of the applied fertiliser combinations on the activity of catalase and peroxidase enzymes and the level of malondialdehyde (MDA) with the drought factor is presented in Table 4. The level of catalase activity in the study was significantly higher in the BS combination compared to the CS combination at all test days. The catalase activity in the CS combination was similar on individual test days, and the lowest value of the tested factor was recorded on day 15. The catalase activity in plant material from the BS combination differed significantly between individual measurement days. In the BS combination, the highest value of catalase activity was recorded on day 12 of the study and the lowest on day 15. The fertiliser combinations used in the experiment influenced the level of MDA in the tested plant material (Table 4). The MDA value in the tested leaves was higher in the BS combination compared to the CS combination on the 2nd and 8th day of measurement, and on the 15th day of measurement, the MDA level was 37% higher in the CS combination compared to the BS combination. In the CS combination, the highest value of the tested indicator was recorded on the 15th day of measurements and the lowest on day 0. In the BS combination, the highest MDA concentration was recorded on the 8th and 15th day of the study, and the lowest on the 2nd and 12th day of the measurements. Peroxidase activities were determined at a similar level between the combinations on test days from 0 to 12, while differences between the combinations were noted only on the 15th day of measurements, when the MDA value was significantly higher in the CS combination compared to BS. In the plant material from the CS combination, the peroxidase activity was similar on individual test days, and the highest value of the tested indicator was recorded on day 0. In the BS combination, the measurements of peroxidase activity differed, the highest values of the tested indicator were recorded on day 0, and the lowest on the 15th day of the study.

**Table 4:** Effect of fertilisation program on the activity of oxidizing enzymes and on the level of the oxidative stress marker malondialdehyde (MDA). Combinations with a drought factor.

	Combination	Sampling days					
		0	2	8	12	15	p-value
Catalase concentration (μmol/min/g-1 FW)	CS	250,4^Ab^	317,2 ^Ab^	298,9^Ab^	252,3^Ab^	199,9^Aa^	0,0004
	BS	292,1^Bb^	357,0^Bcd^	324,8^Bc^	374,2^Bd^	234,0^Ba^	0,0001
	p-value	0,0087	0,0419	0,0428	0,0267	0,0366	
MDA concentration (mg/g-1 FW)	CS	0,032^Aa^	0,053^Ac^	0,043^Ab^	0,042^Ab^	0,097^Bd^	0,0001
	BS	0,055^Bab^	0,053^Aa^	0,056^Bb^	0,048^Aa^	0,061^Ab^	0,1365
	p-value	0,0289	0,4611	0,0709	0,9436	<0,001	
Peroxydase concentration (μmol/min/g^-1^ FW)	CS	0,035^Ab^	0,028^Aa^	0,019^Aa^	0,026^Aa^	0,024^Ba^	0,0030
	BS	0,045^Ac^	0,022^Ab^	0,035^Abc^	0,024^Ab^	0,015^Aa^	0,0164
	p-value	0,8783	0,8102	0,1436	0,3451	0,0164	

Capital letters in the column are for comparing the Modalities, small letters in the line are for comparing the impact of particular fertilisation programmes.

The impact of the fertilisation program (with and without the drought factor) on the nitrogen and carbon content in the leaves of the tested plants is presented in Table 5. The fertiliser combinations used in the experiment did not significantly affect the differences between the tested combinations C, CS, B and BS. In none of the tested combinations were there any differences between individual test days.

**Table 5:** The influence of the fertilisation program on the nitrogen and carbon content in the leaves of the tested plants.

	Combination	Sampling days					
		0	2	8	12	15	p-value
Nitrogen concentration (mg/g-1 DW)	C	21,9^Aa^	22,5^Aa^	24,1^Aa^	22,6 ^Aa^	24,1^Aa^	0,4234
	B	21,6^Aa^	24,2^Aa^	23,9^Aa^	24,4^Aa^	24,6^Aa^	0,6353
	p-value	0,8858	0,3645	0,9833	0,2769	0,8027	
Carbon concentration (mg/g-1 DW)	C	492,1^Aa^	499,5^Aa^	491,2^Aa^	489,7^Aa^	492,9^Aa^	0,2983
	B	492,6^Aa^	495,5^Aa^	490,4^Aa^	488,5^Aa^	498,0^Aa^	0,1150
	p-value	0,9292	0,1943	0,8462	0,7902	0,1179	
Nitrogen concentration	CS	22,9^Aa^	24,2^Aa^	23,3^Aa^	25,2^Aa^	23,4^Aa^	0,4315
	BS	23,2^Aa^	24,3^Aa^	24,9^Aa^	26,7^Aa^	27,5^Aa^	0,3346
	p-value	0,9284	0,9747	0,4348	0,2761	0,225	
(mg/g-1 DW)	CS	496,2^Aa^	495,9^Aa^	492,9^Aa^	497,2^Aa^	490,5^Aa^	0,7723
	BS	489,7^Aa^	486,3^Aa^	495,1^Aa^	489,1^Aa^	491,9^Aa^	0,2152
	p-value	0,101	0,304	0,6861	0,361	0,8703	

Capital letters in the column are for comparing the Modalities, small letters in the line are for comparing the impact of particular fertilisation programmes.

The tests conducted in the experiment on the impact of the fertilisation program on the chlorophyll content in highbush blueberry leaves showed no significant differences between the tested combinations (Table 6). In combination B, the chlorophyll level increased significantly on the 12th and 15th day of measurements. Also in the CS combination, the highest level of chlorophyll in leaves was recorded on the 12th and 15th day of measurements. In Combination C and BS, no significant differences were noted between individual test days.

**Table 6:** The effect of the fertilisation program on the chlorophyll content in the leaves of the tested plants expressed by the leaf colour intensity index SPAD (Soil Plant Analysis Development).

	Combination	Sampling days					
		0	2	8	12	15	p-value
**SPAD**	C	27,3^Aa^	21,3^Aa^	30,6^Aa^	30,1^Aa^	30,6^Aa^	0,0550
	B	17,8^Aa^	24,3^Aa^	23,8^Aa^	31,0^Ab^	36,7^Ab^	0,0198
	p-value	0,8315	0,0532	0,9701	0,5416	0,2461	
	CS	21,9^Aa^	20,0^Aa^	24,4^Aa^	31,6^Ab^	28,2^Ab^	0,0110
	BS	26,2^Aa^	27,9^Aa^	30,4^Aa^	30,5^Aa^	24,2^Aa^	0,8443
	p-value	0,8714	0,7619	0,5779	0,4276	0,5208	

Capital letters in the column are for comparing the Modalities, small letters in the line are for comparing the impact of particular fertilisation programmes.

The impact of the applied fertilisation programs on the accumulation of nutrients in the leaves of the tested plants is presented in Tables 7 and 8.

**Table 7:** The influence of the fertilisation program on the content of minerals in the leaves of the tested plants—Concentration [mg/g-1 DW]. Combinations without the influence of drought.

	Combination	Sampling days					
		0	2	8	12	15	p-value
K	C	5,69 ^Aa^	5,88 ^Aa^	6,79 ^Aa^	7,17^Aa^	6,59^Aa^	0,3399
	B	5,67^Aa^	6,64^Aa^	5,89 ^Aa^	6,86^Aa^	6,48 ^Aa^	0,1796
	p-value	0,9751	0,1556	0,3784	0,5651	0,8721	
Ca	C	7,18 ^Aa^	7,25^Aa^	5,87 ^Aa^	5,94^Aa^	6,01^Aa^	0,2903
	B	6,72 ^Aa^	6,06^Aa^	4,31^Aa^	4,27^Aa^	4,65^Aa^	0,126
	p-value	0,3074	0,399	0,2931	0,442	0,0686	
Mg	C	2,16^Aa^	2,19^Aa^	1,80 ^Aa^	1,85 ^Aa^	1,93 ^Aa^	0,2886
	B	1,96 ^Aa^	1,83^Aa^	1,46 ^Aa^	1,48 ^Aa^	1,34^Aa^	0,0504
	p-value	0,0679	0,0791	0,335	0,1296	0,0566	
P	C	1,85^Aa^	1,78^Aa^	1,79^Aa^	1,81^Aa^	1,66 ^Aa^	0,4641
	B	1,74^Aa^	1,84 ^Aa^	1,81^Aa^	1,85^Aa^	1,77 ^Aa^	0,9139
	p-value	0,5641	0,4813	0,8909	0,6675	0,3411	
B	C	0,065^Aa^	0,06^Aa^	0,05^Aa^	0,06^Aa^	0,06 ^Aa^	0,8273
	B	0,06^Aa^	0,06^Aa^	0,05^Aa^	0,05^Aa^	0,04 ^Aa^	0,2902
	p-value	0,6164	0,3037	0,5974	0,1131	0,0921	
Fe	C	0,27^Aa^	0,32^Aa^	0,35^Aa^	0,36^Aa^	0,34 ^Aa^	0,8405
	B	0,28^Aa^	0,34 ^Aa^	0,32^Aa^	0,34^Aa^	0,22^Aa^	0,8387
	p-value	0,9231	0,8946	0,7533	0,7747	0,2071	
Mn	C	0,17^Aa^	0,18^Aa^	0,17 ^Aa^	0,17^Aa^	0,16^Aa^	0,9893
	B	0,18^Aa^	0,16^Aa^	0,13^Aa^	0,14^Aa^	0,11^Aa^	0,4733
	p-value	0,8247	0,6663	0,4842	0,2901	0,1567	
S	C	3,04 ^Aa^	2,94^Aa^	2,84^Aa^	2,99^Aa^	2,85 ^Aa^	0,9432
	B	2,34^Aa^	2,48^Aa^	2,07^Aa^	2,09^Aa^	1,92^Aa^	0,1373
	p-value	0,368	0,1106	0,172	0,122	0,0655	
Na	C	0,31^Aa^	0,31^Aa^	0,26^Aa^	0,31^Aa^	0,25^Aa^	0,5613
	B	0,25 ^Aa^	0,25 ^Aa^	0,21^Aa^	0,19^Aa^	0,16^Aa^	0,3985
	p-value	0,2297	0,1381	0,3321	0,1023	0,2206	

Capital letters in the column are for comparing the Modalities, small letters in the line are for comparing the impact of particular fertilisation programmes.

**Table 8:** The effect of the fertilisation program on the content of minerals in the leaves of the tested plants—Concentration [mg/g^-1^ DW]. Combinations with a drought factor.

	Combination	Sampling days					
		0	2	8	12	15	p-value
K	CS	5,26^Aa^	5,89^Aa^	5,82^Aa^	6,47^Aa^	6,46^Aa^	0,8187
	BS	6,87^Aa^	7,63^Aa^	7,72^Aa^	8,42^Ab^	9,27^Ab^	0,0407
	p-value	0,1543	0,1696	0,0978	0,0712	0,0615	
Ca	CS	5,49^Aa^	4,94^Aa^	3,98^Aa^	4,58^Aa^	5,11^Aa^	0,3572
	BS	5,45^Aa^	5,42^Aa^	3,96^Aa^	3,61^Aa^	3,96^Aa^	0,1519
	p-value	0,9625	0,4933	0,9807	0,2796	0,3033	
Mg	CS	1,72^Aa^	1,55^Aa^	1,41^Aa^	1,51^Aa^	1,59^Aa^	0,7064
	BS	2,04^Aa^	1,96^Aa^	1,62^Aa^	1,54^Aa^	1,57^Aa^	0,2728
	p-value	0,3241	0,0813	0,5153	0,8617	0,9484	
P	CS	1,74^Aa^	1,81^Aa^	1,79^Aa^	1,72^Aa^	1,51^Aa^	0,3007
	BS	1,74^Aa^	1,82^Aa^	1,87^Aa^	1,94^Aa^	1,84^Aa^	0,9016
	p-value	0,9903	0,9703	0,5352	0,4001	0,0562	
B	CS	0,05^Aa^	0,05^Aa^	0,03^Aa^	0,05^Aa^	0,05^Aa^	0,1345
	BS	0,04^Aa^	0,05^Aa^	0,04^Aa^	0,04^Aa^	0,04^Aa^	0,2563
	p-value	0,3534	0,6887	0,5272	0,3878	0,0571	
Fe	CS	0,12^Aa^	0,22^Aa^	0,25^Aa^	0,25^Aa^	0,31^Aa^	0,0034
	BS	0,21^Aa^	0,32^Aa^	0,31^Aa^	0,26^Aa^	0,27^Aa^	0,3411
	p-value	0,0914	0,1217	0,5667	0,8629	0,6917	
Mn	CS	0,16^Aa^	0,16^Aa^	0,13^Aa^	0,16^Aa^	0,17^Aa^	0,7089
	BS	0,19^Aa^	0,18^Aa^	0,14^Aa^	0,13^Aa^	0,15^Aa^	0,5438
	p-value	0,5892	0,5423	0,7204	0,4077	0,4184	
S	CS	2,04^Aa^	2,12^Aa^	1,83^Aa^	2,02^Aa^	1,97^Aa^	0,6547
	BS	2,49^Aa^	2,60^Aa^	2,19^Aa^	2,18^Aa^	2,16^Aa^	0,7905
	p-value	0,1946	0,1718	0,3684	0,7193	0,6477	
Na	CS	0,22^Aa^	0,25^Aa^	0,18^Aa^	0,23^Aa^	0,27^Aa^	0,8211
	BS	0,32^Aa^	0,34^Aa^	0,24^Aa^	0,24^Aa^	0,29^Aa^	0,3896
	p-value	0,1866	0,3704	0,3571	0,9545	0,7591	

Capital letters in the column are for comparing the Modalities, small letters in the line are for comparing the impact of particular fertilisation programmes.

The tests conducted in the experiment on the accumulation of nutrients in highbush blueberry leaves showed no significant differences between the tested fertiliser combinations. In the experiment carried out, the effect of different fertilisation technologies on the content of 9 nutrients was checked at different days. In both combination C and combination B, there were no significant differences in nutrient content at all test days (Table 7).

The tests conducted in the experiment on the accumulation of nutrients in highbush blueberry leaves showed no significant differences between the tested fertiliser combinations. In the experiment carried out, the effect of different fertilisation technologies on the content of 9 nutrients was checked at different days. The only significant difference was noted in the BS combination, in which the K level significantly increased on the 12th and 15th day of measurements (Table 8).

## Discussion

Drought stress is the main threat negatively affecting crop production in most regions of the world, and its effects are intensifying with climate change [39]. Lowering the level of MDA, H 2 O 2, increasing the activity of oxidizing enzymes and phenolics compounds and ABA (Abscisic Acid) are among the main defence mechanisms of plants during exposure to drought stress [39]. Drought stress significantly reduces the quality and yield of crops, and biostimulants seem to be an effective alternative to eliminating the impact of abiotic stress on plants [15, 16]. The biostimulator used in the experiment is based on marine algae extracts. Seaweed extracts contain organic matter and nutrients, stimulate plant growth, photosynthesis and tolerance to stressors and thus improve the quantity and quality of crops [40]. Catalase and peroxidase are neutralizers of reactive oxygen species (ROS) resulting from drought stress [41]. ROS accumulation in plant cells leads to lipid peroxidation and permanent cell wall damage [42, 43]. As a result of the study, significant accumulation of oxidative stress marker (MDA) was found in plant cells not treated with biostimulation.

Compared to the combination in which the drought factor was not used in the biostimulant combination, the value of this parameter was lower by half. The study confirms the clear positive impact of the tested biostimulator on reducing the level of harmful MDA in plant cells. In the research of Smirnoff N [44] an increase in MDA concentration in sage leaves induced by drought stress was observed. The studies of Mansori M et al. [45], report a positive effect of marine algae extracts on reduction of MDA concentration in the leaves of the tested plants subjected to drought stress. According to numerous studies, biostimulants based on marine algae extracts induce activity of antioxidant enzymes [16, 17, 23, 44]. The results of own research indicated that the level of catalase activity was clearly higher in the combinations in which the stress factor was introduced, and the plants were treated with biostimulation, compared to the control combination with stress factor and without biostimulation. The same relation was observed in combinations where no drought factor was introduced. Plants sprayed with a biostimulator had a higher potential for antioxidant activity than those treated with only nutrients without biostimulation. This relation was not confirmed in the case of peroxidase activity, its level increased with the duration of the experiment and the activity level of this enzyme was similar in the tested combinations. Both catalases and peroxidases are good markers of plant stresses, but it is worth noting that the activity of both catalases and peroxidases in plants may vary depending on the species, environmental conditions, and the type of stress [46]. Numerous scientific studies indicate biostimulants with marine algae extracts as plants supporting preparations in the event of drought stress [47–50]. According to the research of Lola-Luz et al. [51], the use of marine algae extracts increased the activity of oxidoreductases and reduced the accumulation of harmful MDA.

Liu et al. [52] described the positive effect of algae preparations on the resistance of cultivated plants to drought stress by increasing biomass, chlorophyll, and proline content, increasing the activity of antioxidant enzymes, and reducing the MDA content in leaves. Drought stress can cause leaf yellowing through chlorophyll degradation [53]. According to Goña, Quille and O’Connell [54], tomato plants exposed to drought stress and sprayed with marine algae extracts had a higher level of chlorophyll.

The research conducted in this study does not indicate the influence of the biostimulator used on the higher chlorophyll content in leaves (SPAD). The impact of drought stress on the chlorophyll content in leaves is an important aspect of many studies, and the results often depend on the method used and the plant species examined [55]. However, numerous scientific works describe the positive impact of biostimulants from marine algae on the chlorophyll content in various crop species [48, 51, 54, 56, 57]. Sivasankari et al. [58], Mancuso et al. [59] and Spinelli et al. [60] describe in their studies the influence of algae preparations on the increased chlorophyll content in cultivated plants such as grapevine and strawberry. Khan et al. [61], Rathore et al. [62], Roussos et al. [63] report that foliar treatments with marine algae extracts increase the yield and quality of crops in species such as vine, strawberry, tomato and corn. This is related to better nutrition of plants with nutrients [54]. Mancuso et al. [59] showed that the application of seaweed extract on grapevines increased the accumulation of N, P, K, Zn, and Mg. Rathore et al. [62] also showed an increase in the content of N, P, K and S in soybean seeds treated with algal biostimulants. The influence of the examined biostimulator to increase the nutrient content in highbush blueberry plants in greenhouse conditions was not confirmed. Effectiveness of biostimulant preparations depend on several factors, including the environment in which the plants are being grown [63]. There may be differences between field conditions and greenhouse conditions affecting the effectiveness of biostimulants.

The climatic conditions in the greenhouse are controlled, which means that plants are exposed to constant light intensity, temperature, humidity, and other environmental conditions, and are often subjected to only one form of stress, e.g., drought. In field conditions however, plants are more exposed to changing weather conditions, such as varying intensity of sunlight, variable and extreme temperatures, and rainfall. Biostimulants often act as support for plants in the event of stress, so their effects may be more noticeable in more difficult field conditions [64]. This is described in a study conducted by Lenart A et al. [65]. Based on field tests conducted using biostimulation in the cultivation of highbush blueberry, it was shown that fertilisation technology based on algae products resulted in an increase in yield and fruit weight, a high degree of fruit set and high firmness of the tested berries [65].

## Conclusion

Mechanisms that protect against abiotic stress are essential for the survival of plants, and their activation through the use of algal biostimulators is of particular importance for agricultural development. Marine algae extracts can significantly contribute to eliminating the negative effects of stress and building plant resistance, e.g., to stress drought. This was confirmed in the above work. The application of the biostimulant in the experiment resulted in an enhancement of the oxidative activity of peroxidase and catalase enzymes under conditions simulating drought stress. The introduction of the algal biostimulant led to a 20% increase in the average catalase activity compared to the control plants in both experimental groups. While the tested biostimulator effectively stimulated the activity of antioxidant enzymes, it did not exert any influence on the chlorophyll content or nutrient concentration in the leaves. The observed positive effects of marine algae products on yield quantity and quality can be attributed, in part, to their bioactive compounds, which play a role in mitigating drought stress. It’s worth it to note that the activity of mechanisms protecting against abiotic stress in plants may vary depending on species, environmental conditions, and type of stress. It is a part of a more complex plant defence and regulatory system that aims to maintain homeostasis in a changing environment. Usage of plant fertilisation programs with biostimulation create opportunities for producers to produce high-quality crops in a changing climate.

On the basis of the results obtained, greenhouse research on the effect of the biostimulant on drought stress in highbush blueberry should be continued. Studies on the effect of the biostimulant on oxidative enzyme levels, MDA, SPAD and micronutrient content in leaves should be continued, in addition, to better understand the effect of biostimulation during drought stress, studies should be carried out: transcriptomic, proteomic and also paraffin sections of cells.

Permission for research was obtained from Centre Mondial de l’Innovation Roullier to collect plant materials and all study/experimental protocols involving plant materials were conducted in accordance with institutional, national, and international guidelines and legislation.
